# Changes in working status after cancer diagnosis and socio-demographic, clinical, work-related, and psychological factors associated with it

**DOI:** 10.1186/s12885-022-10013-8

**Published:** 2022-08-25

**Authors:** Danbee Kang, Ka Ryeong Bae, Ho Young Kim, Yeojin Ahn, Nayeon Kim, Youngmog Shim, Tae Sung Sohn, Woo Yong Lee, Ji Hyun Baek, Sun-Seog Kweon, Juhee Cho

**Affiliations:** 1 Department of Clinical Research Design and Evaluation, Samsung Advanced Institute for Health Sciences and Technology, Sungkyunkwan University, Seoul, South Korea; 2 Center for Clinical Epidemiology, Samsung Medical Center, Seoul, South Korea; 3 National Cancer Survivorship Center, National Cancer Control Institute, National Cancer Center, Goyang, South Korea; 4 Cancer Education Center, Samsung Medical Center, Seoul, South Korea; 5 Department of Thoracic Surgery, Samsung Medical Center, Sungkyunkwan University School of Medicine, Seoul, South Korea; 6 Department of Surgery, Samsung Medical Center, Sungkyunkwan University School of Medicine, Seoul, South Korea; 7 Gwangju-Jeonnam Regional Cancer Center, Chonnam National University Hwasun Hospital, Hwasun, South Korea; 8 Department of Preventive Medicine, Chonnam National University Medical School, Gwangju, South Korea

**Keywords:** Cancer survivors, Work, Employment, Unemployment, Return to work, Cross-sectional studies, Korea

## Abstract

**Background:**

While many studies investigated changes in working status in cancer survivors, most studies have been performed in survivors of breast cancer and few studies evaluated factors associated with changes in the working status of cancer survivors comprehensively. We aimed to evaluate the changes in the working status of cancer survivors after diagnosis and socio-demographic, clinical, work-related and psychological factors associated with it.

**Methods:**

We conducted a cross-sectional survey of adult patients with cancer who were working at the time of diagnosis. A trained interviewer inquired about participants’ current working status, including leave of absence, discontinuing, continuing, and changing work. Sociodemographic, clinical, work-related and psychological factors were measured. Multinomial logistic regression was used to identify factors associated with changes in the working status.

**Results:**

Among the 730 patients, 29%, 18% and 6% were currently on a discontinued working, leave of absence and had changed jobs, respectively. Patients who discontinued working after cancer diagnosis were more likely to be female, have ≥ $3,000 of monthly family income, not be the principal wage earners for their families and be blue-collar workers. In clinical characteristics, advanced-stage cancer and experienced cancer recurrence was associated with leave of absence and discontinued working. In work-related and psychological factors, stress due to insufficient job control (relative risk ratio [RRR] = 2.26), interpersonal conflict (RRR = 1.86), job insecurity (RRR = 2.63), organizational system (RRR = 3.49), and lack of reward (RRR = 11.76), and less meaning to work were more likely to discontinue working after a cancer diagnosis.

**Conclusion:**

Occupational health care professionals and other stakeholders need to openly communicate with patients with cancer about potential barriers during the return-to-work trajectory.

**Supplementary Information:**

The online version contains supplementary material available at 10.1186/s12885-022-10013-8.

## Introduction

Working after a cancer diagnosis is often associated with returning to normalcy for patients [1]. From a personal perspective, this allows patients with cancer and their families to maintain their economic well-being through a steady income from paid work. Furthermore, it can promote psychological stability as a source of maintaining identity and self-realization [2]. From a social perspective, working patients with cancer are valuable as they help develop and maintain socially productive human resources [3]. Moreover, working after cancer diagnosis and treatment is an important milestone in the transition period, especially for those of working age [4]. More than half of patients with cancer are of working age, and as the retirement age rises in many countries, the number of working patients with cancer is increasing [5, 6].

However, the unemployment rate in patients with cancer is 1.37 times higher than that in healthy individuals [6]. According to previous studies, 26–50% of patients with cancer discontinued working after their diagnosis, and 23–75% of those who discontinued working were re-employed [7, 8]. Various factors can cause or increase the risk of discontinuing work after cancer diagnoses [6]. Regarding personal factors, old age, low income, and being female significantly increased the risk of patients with cancer to discontinue working [8, 9]. In terms of clinical factors, cancer site, physical symptoms, and an unfavorable prognosis were associated with discontinuing work [7]. Perceived employer discrimination and manual work were associated with discontinuing work after cancer diagnosis [7]. Especially, discrimination was significantly correlated with forced unemployment [9]. Recently, the importance of employment factors, such as an individual’s meaning of work and relationships with coworkers and managers, has been highlighted [10, 11].

While many studies investigated changes in working status in cancer survivors, few studies evaluated factors associated with changes in the working status of cancer survivors comprehensively. Most studies have been performed on survivors of breast cancer [12, 13] or qualitative method [14]. In addition, previous literature has been limited to patients in Western countries, although factors related to working status may have cultural specificity [12, 13]. Thus, we aimed to evaluate the changes in the working status of cancer survivors after diagnosis and socio-demographic, clinical, work-related and psychological factors associated with it.

## Materials and methods

### Participants

We conducted a cross-sectional survey of adult patients with cancer who were working at the time of diagnosis. Patients with cancer were recruited from outpatient clinics or inpatient wards at the Samsung Medical Center in Seoul and the Chonnam National University Hospital in Jeolla-do, Korea, from October 2017 to March 2018. Patients were eligible if they were aged 20–65 years old, if they were working at the time of diagnosis, and if they had anti-cancer treatment with curative intent. At the outpatient clinics and inpatients wards, clinicians introduced the study to eligible patients. Once patients agreed to participate in the study and signed the informed consent, trained researchers explained the purpose and procedures of the study. Then, participants were asked to complete the survey on paper. The study was approved by the Institutional Review Boards of Samsung Medical Center (IRB 2017–05-166) and Chonnam National University Hospital (IRB TMP-2017–118).

### Measures

A trained interviewer asked study participants about changes in working status after cancer diagnosis (continued to work, leave of absence, discontinuing, or changing work). For participants who discontinued working, we asked additional questions regarding the time at which they discontinued working (at diagnosis, before treatment, during treatment, or after treatment). Job type was defined as white-collar (legislators, senior officials, managers, professionals, technicians, and associate professionals), blue-collar (skilled agricultural and fishery workers, craft and related trade workers, plant and machine operators and assemblers, workers in elementary occupations, and armed forces occupations), or service or sales (clerks and service workers and shop and market sales workers) according to the International Standard Classification of Occupations [15].

Regarding work-related and psychological factors, we measured occupational stress and meaning in work, respectively. To assess occupational stress, we used the standardized Korean Occupational Stress Scale (KOSS), which is unique and specific to occupational stressors among Korean employees. The KOSS comprises 24 questions in seven domains: job demand, insufficient job control, interpersonal conflict, job insecurity, organizational system, lack of reward, and occupational climate. The items were scored on a conventional Likert scale (ranging from 1 to 4) for the response categories. The KOSS domains were scored according to the published scoring manual [16], and the data were linearly transformed to yield scores from 0 to 100. To perform the analysis, we divided the reference values, which were the medians of the general Korean population (KOSHA GUIDE H-67–2012).

Meaning in work was evaluated using the Work and Meaning Inventory (WAMI) [17, 18]. The WAMI contains 10 items that are measured using a 5-point Likert scale ranging from 1 (totally disagree) to 5 (totally agree). It assesses three dimensions of meaning in work: positive meaning, meaning-making through work, and greater good motivations. Low scores on any of these scales reflect an absence of meaningful work and may be predictive of poor work engagement, low commitment to one’s organization and intention to leave, low motivation, and a perceived lack of support and adequate guidance from leadership or management.

The participants were also asked about clinical factors, such as the primary cancer site, clinical stage, time since diagnosis, and experience of recurrence, and sociodemographic factors, including sex, age, marital status, education, and current and principal wage earners.

### Statistical analysis

The analyses were conducted for four groups of participants, including those who were granted leave of absence, discontinued work, continued to work, or changed jobs. Descriptive statistics were used to summarize the sociodemographic characteristics of participants and factors associated with work status. To compare the groups, we used the chi-square test and analysis of variance for categorical and continuous outcomes, respectively.

Multinomial logistic regression was performed to identify the factors associated with current working status, and continued working was used as the reference group. The relative risk ratios (RRRs) and 95% confidence intervals (CIs) were calculated. Furthermore, based on the manual, the stress score was divided into dichotomous variables according to the occupational stress score in the normal group [16]. Meaning in work was also divided dichotomously, according to the median of the study distribution. In the multinomial logistic regression, we adjusted for age, sex, cancer type, cancer stage, time since diagnosis, and experience of cancer recurrence. All statistical analyses were performed using Stata 15 (StataCorp LP, College Station, TX, USA), and *p*-values were two-sided. Statistical significance was set at *p* < 0.05.

## Results

**Fig. 1:**
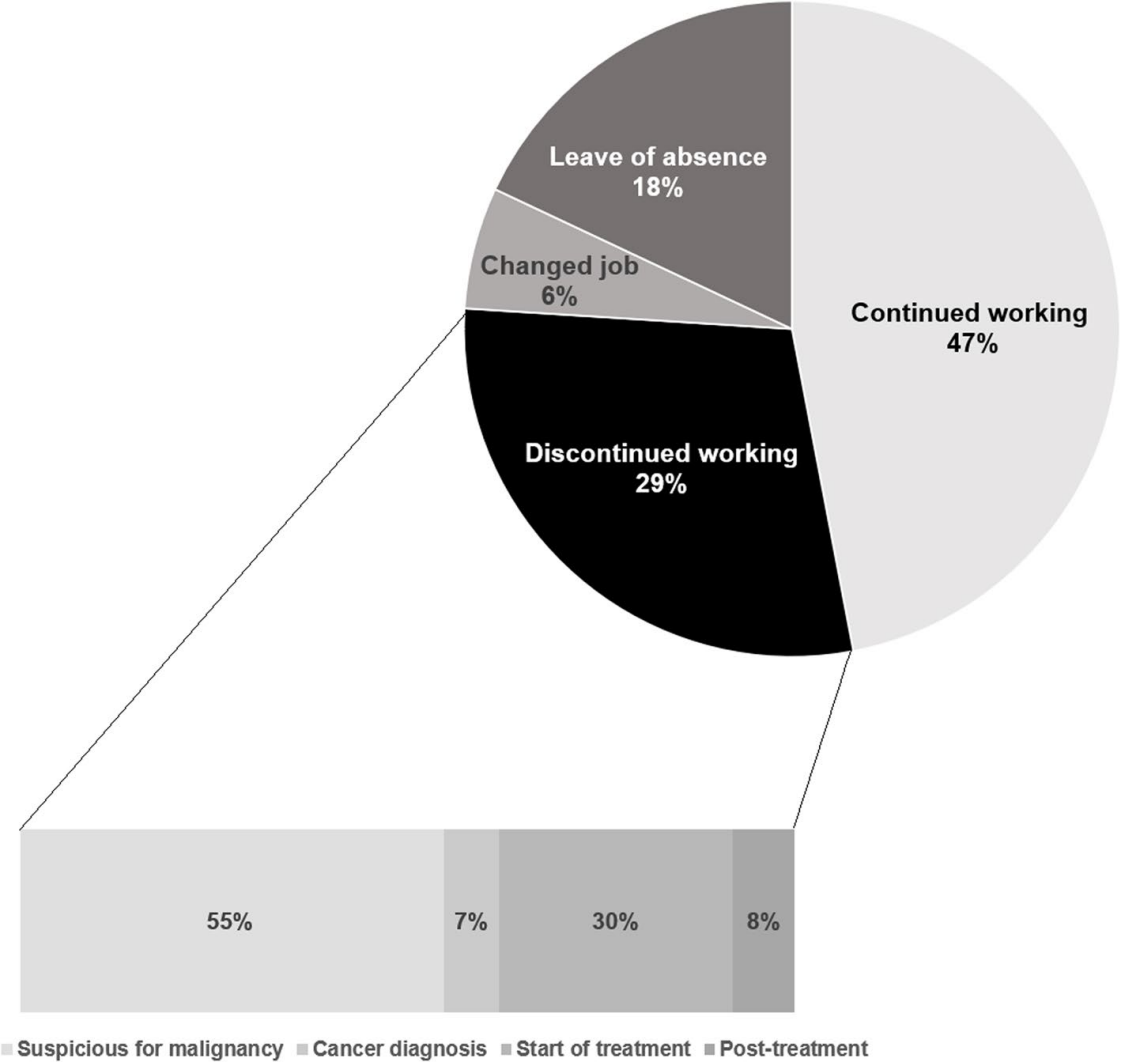
Changes in working status after cancer diagnosis (*N* = 730)

A total of 730 patients participated and completed the survey according to the instructions. The study participants’ mean (standard deviation) age at the time of the survey was 52.7 (8.8) years, 55.2% of the participants were male, and the time since diagnosis was 22.8 (21.7) months. In terms of working status, 47% (*N* = 340) of the participants continued to work, and 29% (*N* = 211) discontinued working (Fig. 1). Among those who discontinued working, 55% quit immediately after the cancer diagnosis. Furthermore, 18% and 6% were currently on a leave of absence and had changed jobs, respectively. Patients who discontinued working after cancer diagnosis were more likely to be female, have ≥ $3,000 of monthly family income, and not be the principal wage earners for their families. Moreover, this group had the lowest proportion of white-collar workers (Table 1). In terms of clinical characteristics, patients who discontinued working were more likely to have advanced-stage cancer and experienced cancer recurrence (Table 1).

**Table 1:** Characteristics of the study participants (*N* = 730)

	**Leave of absence**	**Discontinued working**	**Continued working**	**Changed job**	***P*-value**
	**(*N* = 134)**	**(*N* = 211)**	**(*N* = 340)**	**(*N* = 45)**	
**Demographic factors**					
**Age (years), mean (*SD***^a^**)**	49.9 (8.8)	53.5 (8.9)	53.2 (8.5)	52.6 (8.9)	0.001
**Sex, male**	55 (41.0)	71 (33.6)	246 (72.4)	31 (68.9)	< 0.001
**Marital status, married**	110 (82.1)	171 (81.0)	303 (89.1)	33 (73.3)	< 0.001
**Monthly family income**					< 0.001
< $3,000	51 (38.1)	118 (55.9)	71 (20.9)	18 (40.0)	
≥ $3,000	81 (60.4)	79 (37.4)	266 (78.2)	26 (57.8)	
Unknown	2 (1.5)	14 (6.6)	3 (0.9)	1 (2.2)	
**Principal wage earner**					< 0.001
Patient alone	46 (34.3)	49 (23.2)	203 (59.7)	27 (60.0)	
Patient and other family members	21 (15.7)	13 (6.2)	82 (24.1)	9 (20.0)	
Other family members	63 (47.0)	142 (67.3)	52 (15.3)	9 (20.0)	
Unknown	4 (3.0)	7 (3.3)	3 (0.9)	0 (0)	
**Education**					0.14
< high school	15 (11.2)	37 (17.5)	32 (9.4)	4 (8.9)	
≥ high school	119 (88.8)	173 (82)	307 (90.3)	41 (91.1)	
Unknown	0 (0)	1 (0.5)	1 (0.3)	0 (0)	
**Job type at diagnosis**					0.019
White collar	83 (61.9)	118 (55.9)	235 (69.1)	27 (60.0)	
Service or sales	24 (17.9)	49 (23.2)	39 (11.5)	9 (20.0)	
Blue collar	26 (19.4)	44 (20.9)	66 (19.4)	9 (20.0)	
**Clinical factors**					
**Cancer type**					< 0.001
Breast cancer	58 (43.3)	85 (40.3)	51 (15.0)	7 (15.6)	
Pancreatobiliary cancer	9 (6.7)	13 (6.2)	49 (14.4)	9 (20.0)	
Lung/esophagus cancer	26 (19.4)	25 (11.8)	75 (22.1)	8 (17.8)	
Urologic cancer	6 (4.5)	8 (3.8)	87 (25.6)	9 (20.0)	
Colorectal cancer	10 (7.5)	26 (12.3)	19 (5.6)	2 (4.4)	
Gastric cancer	5 (3.7)	12 (5.7)	17 (5.0)	1 (2.2)	
Liver cancer	5 (3.7)	8 (3.8)	14 (4.1)	3 (6.7)	
Others	15 (11.2)	34 (16.1)	28 (8.2)	6 (13.3)	
**Disease stage at diagnosis**					< 0.001
Stage I	38 (28.4)	50 (23.7)	146 (42.9)	16 (35.6)	
Stage II	38 (28.4)	63 (29.9)	86 (25.3)	11 (24.4)	
Stage III	33 (24.6)	57 (27.0)	47 (13.8)	6 (13.3)	
Stage IV	16 (11.9)	19 (9.0)	16 (4.7)	1 (2.2)	
Unknown	9 (6.7)	22 (10.4)	45 (13.2)	11 (24.4)	
**Experienced cancer recurrence**	30 (22.4)	59 (28.0)	48 (14.1)	8 (17.8)	0.001
**Time since diagnosis (months)**	7.0 (3.5–13.1)	14.4 (6.0–31.2)	20.7 (8.5–85.1)	37.4 (27.6–50.4)	< 0.001

Values are presented as number (%), mean (SD), or median (interquartile range)

^a^*SD* Standard deviation

Occupational stress at the time of diagnosis was the highest in patients who discontinued working (Table 2). This group of survivors reported a much higher level of stress due to insufficient job control, interpersonal conflict, the organizational system, lack of reward, and the occupational climate than the other groups. Stress due to job insecurity was high among the participants who discontinued working or changed jobs (Table 2). Furthermore, among the four groups, the group that discontinued working reported the lowest scores on psychological factors pertaining to meaningful work (Table 2). The discontinued-working group reported the lowest positive meaning and the lowest meaning-making through work (Table 2).

**Table 2:** Occupational stress and meaning of work by working status after cancer diagnosis (*N* = 730)

	**Leave of absence**	**Discontinued working**	**Continued working**	**Changed job**	***P*-value**
	**(*N* = 134) Mean (*SD***^a^**)**	**(*N* = 211) Mean (*SD*)**	**(*N* = 340) Mean (*SD*)**	**(*N* = 45) Mean (*SD*)**	
**Occupational stress**^b^					
Job demand (*N* = 666)	46.3 (11.8)	46.1 (14.1)	45.7 (11.2)	46.9 (9.8)	0.93
Insufficient job control (*N* = 662)	47.6 (17.9)	53.5 (23.7)	39.3 (19.9)	43.5 (25.1)	< 0.01
Interpersonal conflict (*N* = 607)	38.8 (19.2)	49.7 (25.0)	39.1 (21.9)	41.7 (20.1)	< 0.01
Job insecurity (*N* = 621)	33.6 (27.4)	35.8 (27.0)	27.2 (23.9)	34.1 (27.4)	< 0.01
Organizational system (*N* = 596)	43.8 (16.6)	56.3 (23.8)	40.7 (19.4)	42.3 (14.0)	< 0.01
Lack of reward (*N* = 618)	41.4 (17.6)	46.2 (24.7)	33.4 (18.2)	34.7 (14.9)	< 0.01
Occupational climate (*N* = 596)	34.7 (18.3)	34.8 (21.1)	29.9 (17.4)	32.5 (19.7)	0.03
**Meaning of work**^c^					
Positive meaning	11.0 (2.7)	9.6 (2.9)	11.5 (2.3)	11.1 (2.7)	< 0.01
Meaning-making through work	10.6 (2.8)	9.5 (3.1)	11.2 (2.5)	10.6 (3.2)	< 0.01
Greater good motivations	11.2 (2.7)	10.0 (2.7)	11.4 (2.6)	11.5 (2.9)	< 0.01

^a^*SD* Standard deviation

^b^Higher scores indicate more stress due to work when the patients had worked (range 0–100)

^c^Higher scores indicate obtaining more value from work (range 5–15)

The fully adjusted RRRs (95% CI) for discontinued working, leave of absence and changed job in females were 3.65 (2.09, 6.38), 1.63 (0.83, 3.16), and 1.07 (0.38, 3.00), respectively (Table 3). When other family members were the principal wage earners, the RRR for discontinued working (RRR = 6.76; 95% CI = 4.06, 11.26) and leave of absence (RRR = 3.52; 95% CI = 2.00, 6.20) was higher than when the patients themselves were principal wage earners (Table 3). Furthermore, blue-collar work (RRR = 1.84; 95% CI = 1.07, 3.15) was associated with work cessation. In terms of clinical factors, an advanced cancer stage and experience of cancer recurrence were risk factors for discontinuing work and leave of absence. In addition, we found that the number of patients who leave of absence decreased according to longer survivors. However, the proportion of patients who discontinued working was similar during all the survivorship period (Table 3). Regarding work-related factors, there was a higher level of stress than the median of the general Korean population in terms of insufficient job control (RRR = 2.26; 95% CI = 1.38, 3.71), interpersonal conflict (RRR = 1.86; 95% CI = 1.18, 2.92), job insecurity (RRR = 2.63; 95% CI = 1.44, 4.82), organizational system (RRR = 3.49; 95% CI = 2.12, 5.72), and lack of reward (RRR = 11.76; 95% CI = 3.28, 42.1). Moreover, attaining less meaning in work than the median of our study distribution was related to discontinuing work after diagnosis (Table 3). When we performed subgroup analysis by time since diagnosis, patients who did not marry, who thought another member is the head of the household, and who had lower work motivations were stronger risk factors in patients who were diagnosed within 24 months compared to those in the patient who survived > 24 months (supplement Table 1).

**Table 3:** Factors associated with changes in working status after cancer diagnosis (*N* = 730)

	**Discontinued working**	**Leave of absence**	**Changed job**
	**RRR**^a^**(95% CI**^b^**)**	**RRR (95% CI)**	**RRR (95% CI)**
**Demographic factors**			
**Age (years)**	1.05 (1.02, 1.08)	0.99 (0.97, 1.02)	0.99 (0.95, 1.03)
**Sex, female**	**3.65 (2.09, 6.38)**	1.63 (0.84, 3.16)	1.07 (0.38, 3.00)
**Marital status, not married**	1.62 (0.91, 2.87)	1.23 (0.64, 2.35)	**3.36 (1.42, 7.95)**
**Education, < high school**	1.67 (0.89, 3.12)	1.60 (0.74, 3.44)	1.02 (0.32, 3.24)
**Principal wage earner**			
Patient alone	*Reference*	*Reference*	*Reference*
Patient and other family members	0.53 (0.26, 1.08)	0.97 (0.50, 1.85)	0.71 (0.31, 1.65)
Other family members	**6.76 (4.06, 11.26)**	**3.52 (2.00, 6.20)**	0.99 (0.39, 2.47)
**Job type at diagnosis**			
White collar	*Reference*	*Reference*	*Reference*
Service or sales	1.28 (0.73, 2.23)	1.33 (0.69, 2.55)	2.17 (0.87, 5.45)
Blue collar	**1.84 (1.07, 3.15)**	1.65 (0.89, 3.05)	1.19 (0.50, 2.85)
**Clinical factors**			
**Cancer type**			
Breast cancer	**8.18 (3.01, 22.25)**	**8.01 (2.53, 25.39)**	2.01 (0.46, 8.81)
Pancreatobiliary cancer	2.71 (0.98, 7.54)	**3.81 (1.15, 12.58)**	1.61 (0.56, 4.62)
Lung/esophagus cancer	**3.38 (1.36, 8.36)**	**4.75 (1.74, 12.96)**	1.39 (0.48, 4.00)
Urologic cancer	*Reference*	*Reference*	*Reference*
Colorectal cancer	**12.47 (4.55, 34.15)**	**4.88 (1.46, 16.31)**	1.41 (0.27, 7.50)
Gastric cancer	**6.50 (2.10, 20.13)**	2.68 (0.68, 10.65)	1.01 (0.11, 9.21)
Liver cancer	**5.43 (1.65, 17.87)**	**5.55 (1.34, 22.92)**	1.92 (0.43, 8.47)
Others	**9.89 (3.69, 26.56)**	**4.40 (1.41, 13.74)**	2.21 (0.57, 8.55)
**Disease stage at diagnosis**			
Stage I	*Reference*	*Reference*	*Reference*
Stage II	1.67 (0.98, 2.84)	1.20 (0.67, 2.18)	1.11 (0.47, 2.64)
Stage III	**3.18 (1.79, 5.66)**	**2.11 (1.11, 3.99)**	1.32 (0.47, 3.75)
Stage IV	2.00 (0.83, 4.79)	2.37 (0.96, 5.87)	0.71 (0.08, 6.06)
**Experienced cancer recurrence**	**2.48 (1.45, 4.24)**	**2.81 (1.51, 5.23)**	1.03 (0.43, 2.51)
**Time since diagnosis (months)**			
< 12	1.23 (0.70, 2.16)	**7.18 (3.21, 16.07)**	0.12 (0.04, 0.37)
12 to < 24	1.80 (0.98, 3.30)	**3.80 (1.58, 9.13)**	0.34 (0.13, 0.90)
24 to < 36	0.76 (0.38, 1.56)	0.73 (0.22, 2.37)	0.65 (0.28, 1.48)
≥ 36	*Reference*	*Reference*	*Reference*
**Work-related and psychological factors**			
**Occupational stress**			
Job demand	1.21 (0.73, 2.01)	1.44 (0.82, 2.51)	1.23 (0.54, 2.82)
Insufficient job control	**2.26 (1.38, 3.71)**	1.14 (0.63, 2.08)	1.72 (0.81, 3.65)
Interpersonal conflict	**1.86 (1.18, 2.92)**	0.64 (0.38, 1.09)	0.78 (0.38, 1.59)
Job insecurity	**2.63 (1.44, 4.82)**	**2.43 (1.23, 4.81)**	1.78 (0.73, 4.37)
Organizational system	**3.49 (2.12, 5.72)**	1.18 (0.66, 2.11)	0.79 (0.33, 1.88)
Lack of reward	**11.76 (3.28, 42.1)**	3.36 (0.68, 16.52)	2.03 (0.21, 19.83)
Occupational climate	1.53 (0.87, 2.69)	0.96 (0.49, 1.88)	1.20 (0.48, 2.99)
**Meaning of work**			
Positive meaning, ≤ 11	**4.49 (2.81, 7.18)**	1.53 (0.96, 2.46)	0.88 (0.45, 1.70)
Meaning-making through work, ≤ 11	**3.50 (2.23, 5.51)**	1.49 (0.93, 2.38)	1.05 (0.54, 2.04)
Greater good motivations, ≤ 11	**3.01 (1.94, 4.66)**	1.38 (0.86, 2.21)	(0.37, 1.40)

Adjusted for age, sex, cancer type, cancer stage, time since diagnosis, and experience of cancer recurrence

^a^*RRR* Relative risk ratios

^b^*CI* Confidence intervals

## Discussion

In this study, one-third of patients with cancer discontinued working, and half of them quit immediately after diagnosis. This might be due to a misunderstanding or lack of knowledge about cancer. Patients might have quit working immediately after the diagnosis due to their belief that they would not be able to work because of cancer [7, 19]. Moreover, they might have quit working as they believed that they would remain unhealthy or be inactive after cancer treatment. According to a recent study, patients with cancer who had negative attitudes toward recovery were about 3 times more likely to discontinue working than those who did not [20]. Thus, it is necessary to educate patients with cancer that they can continuing work even after cancer diagnosis to prevent unemployment due to cancer stigma.

Discontinuing work was associated with unfavorable working environments and a lack of systematic support in the overall period. Patients with cancer who discontinued work experienced higher stress related to job demand, interpersonal conflict, organizational system, lack of rewards, and occupational climate. Individual efforts to reduce job stress are required, but interventions from management and organizations are also needed to reduce interpersonal conflict and lack of rewards at the national level [21]. More importantly, individuals should not be discriminated against because they are patients with cancer. Occupational climate that recognizes and rewards cancer patients according to their abilities would be necessary. It is necessary to emphasize the value of work for patients with cancer and educate them regarding its benefits [22] considering that patients with cancer who perceived their work to be less meaningful were more likely to discontinue working.

Socio-demographic was associated with discontinued working in a short-term period. In our study, being female, having advanced-stage cancer at diagnosis, and doing blue-collar work was associated with discontinuing work after cancer diagnosis. This finding is similar to those of previous studies. Women were less likely to work after cancer diagnosis than men [7, 23]. This result might be related to the traditional notion that men earn money, while women mainly raise children, support the elderly, and perform household duties [24]. Cancer and its treatment can cause a variety of physical symptoms that may adversely affect patients’ ability to work [25, 26] and advanced-stage cancer or recurrence could pose more difficulties in returning to work. In this study, patients with colorectal cancer were more likely to discontinue working than those with other types of cancers. Discomfort due to leakage of the colostomy bag, skin irritation, and peripheral neuropathy caused by chemotherapy also make it difficult for patients with colon cancer to work [27]. Service, sales, and blue-collar workers were more likely to discontinue working than white-collar workers. This is because these types of jobs require physical tasks such as heavy lifting and bending. In previous studies, physical demands of a job were associated with unemployment in patients with cancer [25, 28]. Therefore, a multidisciplinary rehabilitation program that combines occupational counseling with a supervised physical exercise program is likely to facilitate return to work, reduce fatigue, and increase importance of work, work ability, and quality of life [29].

However, patients who reported that they were the principal wage earners for their families were more likely to continue to work than those who were not. Economically vulnerable groups were more likely to continue working instead of resting and focusing on their treatment [7, 26]. Although some patients with cancer wanted to take a leave of absence during treatment, they had to continue working for affording treatment costs or keeping their health insurance [22]. Furthermore, some patients with cancer had to return to work without fully recovering because of financial difficulties in their households, resulting in a deterioration in their health or exposing them to greater risk; which might place a greater financial burden on their families in the future [30]. Considering the increasing cancer survivorship rate and its financial impact, governments and companies should develop a system for helping working cancer patients so that they can focus on their treatment without having financial concerns.

Meaning of work was associated with discontinued working in a long-term period. Finding meaning in work is important for patients with cancer as it provides a sense of identity, normalcy, and fairness [4]. As circumstances change after being diagnosed with cancer, it affects patients’ ability to resume work physically and mentally, making them feel uncertain about their work; thus, as these changes occur, the meaning of work for patients with cancer diminishes [31].

This study had several limitations. First, data on changes in survivors’ employment status over time were based on self-report. Some participants’ responses might have been influenced by social desirability. They could have either under-report or overreport regarding their working status and problems at the workplace. However, to minimize potential bias, we used standardized questionnaires. Second, recall bias might have influenced the results as patients who lost their jobs were more likely to recall job-related stress than those who did not. Furthermore, negative memory biases might also be exacerbated following traumatic events, such as a cancer diagnosis [25, 32]. Although we included a patient who had various survival time periods between < 1 year and more than 5 years after diagnosis and performed subgroup analysis by survival period, further study is needed as prospective methods. Third, as we recruited study participants from two large cancer centers in Korea, the results might not be generalizable to patients at other settings. Yet, we conducted the study at one hospital each in Seoul and a rural area in Korea. We also included patients with various cancer types, ages, and occupations.

Despite these limitations, this study examined a relatively large number of working-age cancer survivors and evaluated their working status after cancer diagnosis and including various factors associated with employment. While further longitudinal studies are necessary to confirm these findings, the results of this study suggest that cancer survivors need to be informed about possible work-related changes from diagnosis. It also emphasizes the necessity of public campaigns to reduce stigma toward cancer and patients with cancer considering that many cancer survivors experience discrimination at the workplace. Occupational healthcare professionals and other stakeholders need to openly communicate about potential barriers during the return-to-work trajectory and develop a system for working-age cancer patients.

## Supplementary Information

**Additional file 1:Supplement Table 1.** Factors associated with discontinued working after cancer diagnosis by time since diagnosis (N = 533).

## Data Availability

The datasets generated during and/or analysed during the current study are available from the corresponding author (JC) on reasonable request.
