# Supplementary material for: Changes in working status after cancer diagnosis and socio-demographic, clinical, work-related, and psychological factors associated with it
Source: BMC Cancer. 2022 Aug 25;22:917. doi: 10.1186/s12885-022-10013-8 (PMC9404600; doi:10.1186/s12885-022-10013-8)
Supplement: Supplementary file 1 — Additional file 1:Supplement Table 1. Factors associated with discontinued working after cancer diagnosis by time since diagnosis (N = 533). [file 12885_2022_10013_MOESM1_ESM.docx]

**Supplement Table 1. Factors associated with discontinued working after cancer diagnosis by time since diagnosis (N = 533)**

|  |  |  | **≤24 months (N = 443)** | **>24 months (N = 443)** |
| --- | --- | --- | --- | --- |
|  |  |  | **OR (95% CI)** | **OR (95% CI)** |
| **Demographic factors** | | |  |  |
|  | **Age (years)** | | 1.03 (1, 1.06) | 1.09 (1.03, 1.14) |
|  | **Sex, female** | | **4.86 (2.26, 10.45)** | **3.79 (1.2, 11.99)** |
|  | **Marital status, no married** | | **3.06 (1.39, 6.76)** | 1 (0.31, 3.19) |
|  | **Education, < high school** | | **2.75 (1.13, 6.69)** | 1.39 (0.46, 4.2) |
|  | **Principal wage earner** | |  |  |
|  |  | Patient alone | *Reference* | *Reference* |
|  |  | Patient and other family member | 0.37 (0.14, 0.99) | 1.3 (0.36, 4.66) |
|  |  | Other family member | **3.79 (1.93, 7.43)** | **31.71 (9.26, 108.59)** |
|  | **Type of job at diagnosis** | |  |  |
|  |  | White-Collar | *Reference* | *Reference* |
|  |  | Service or sales | 2.1 (0.97, 4.56) | 0.69 (0.23, 2.11) |
|  |  | Blue-Collar | 1.84 (0.89, 3.79) | 1.25 (0.47, 3.3) |
| **Clinical factors** | | |  |  |
|  | **Cancer type** | |  |  |
|  |  | Breast cancer | **4.87 (1.22, 19.43)** | **27.81 (4.4, 175.73)** |
|  |  | Pancreatobiliary cancer | 2.41 (0.56, 10.44) | **5.81 (1.1, 30.65)** |
|  |  | Lung/esophagus cancer | 2.51 (0.69, 9.1) | **6.86 (1.38, 34.09)** |
|  |  | Urologic cancer | *Reference* | *Reference* |
|  |  | Colorectal cancer | **11.27 (2.84, 44.62)** | **32.27 (5.24, 198.65)** |
|  |  | Gastric cancer | 2.33 (0.41, 13.15) | **50.49 (6.27, 406.38)** |
|  |  | Liver cancer | **9.9 (1.73, 56.63)** | **1.53 (0.17, 13.52)** |
|  |  | Others | **11.54 (3.05, 43.64)** | **5.65 (0.81, 39.39)** |
|  | **Disease stage at diagnosis** | |  |  |
|  |  | Stage I |  |  |
|  |  | Stage II | **2.23 (1.1, 4.5)** | 1.05 (0.38, 2.89) |
|  |  | Stage III | **3.81 (1.73, 8.42)** | 1.19 (0.4, 3.53) |
|  |  | Stage IV | 2.45 (0.81, 7.44) | 1.61 (0.25, 10.26) |
|  | **Experienced cancer recurrence** | | 1.66 (0.73, 3.76) | **5.18 (2.05, 13.09)** |
| **Work-related and psychological factors** | | |  |  |
|  | **Occupational stress** | |  |  |
|  |  | Job demand | 1.32 (0.69, 2.54) | 0.72 (0.24, 2.2) |
|  |  | Degree of autonomy | 1.8 (0.92, 3.52) | 2.43 (0.96, 6.17) |
|  |  | Relationship conflict | **2.35 (1.24, 4.43)** | **2.49 (1.03, 6.03)** |
|  |  | Job instability | **2.52 (1.06, 5.96)** | **5.28 (1.65, 16.94)** |
|  |  | Organizational system | **3.33 (1.7, 6.54)** | **4.86 (1.81, 13.06)** |
|  |  | Lack of reward | **13.1 (1.61, 106.94)** | **7.25 (1.09, 48.31)** |
|  |  | Occupational climate | 1.87 (0.85, 4.11) | 2.05 (0.67, 6.21) |
|  | **Meaning of the work** | |  |  |
|  |  | Positive meaning, ≤11 | 0.88 (0.45, 1.7) | 1.53 (0.96, 2.46) |
|  |  | Meaning-making through work, ≤11 | 1.05 (0.54, 2.04) | 1.49 (0.93, 2.38) |
|  |  | Greater good motivations, ≤11 | **1.92 (1.07, 3.46)** | **7.16 (2.76, 18.55)** |

P for interaction of time since diagnosis were statistically significant in marital status (P = 0.017), head of the household (P = 0.027), and greater good motivations (P = 0.034).
